# Supplementary material for: ROCOv2: Radiology Objects in COntext Version 2, an Updated Multimodal Image Dataset
Source: Sci Data. 2024 Jun 26;11:688. doi: 10.1038/s41597-024-03496-6 (PMC11208523; doi:10.1038/s41597-024-03496-6)
Supplement: Supplementary file 1 — Supplementary Information [file 41597_2024_3496_MOESM1_ESM.pdf]

ROCOv2: Radiology Objects in COntext Version 2, an Updated Multimodal Image Dataset

Johannes Rückert[1], Louise Bloch[1,2,3,†], Raphael Brüngel[1,2,3,†], Ahmad Idrissi-Yaghir[1,2,†], Henning Schäfer[1,4,†], Cynthia S. Schmidt[3,4,†], Sven Koitka[3,5], Obioma Pelka[1,2,3], Asma Ben Abacha[6], Alba G. Seco de Herrera[7], Henning Müller[8], Peter A. Horn[4], Felix Nensa[3,5], and Christoph M. Friedrich[1,2,*]

[1]Department of Computer Science, University of Applied Sciences and Arts Dortmund, Dortmund, Germany
[2]Institute for Medical Informatics, Biometry and Epidemiology (IMIBE), University Hospital Essen, Essen, Germany
[3]Institute for Artificial Intelligence in Medicine (IKIM), University Hospital Essen, Essen, Germany
[4]Institute for Transfusion Medicine, University Hospital Essen, Essen, Germany
[5]Institute of Diagnostic and Interventional Radiology and Neuroradiology, University Hospital Essen, Essen, Germany
[6]Microsoft, Redmond, Washington, USA
[7]University of Essex, Wivenhoe Park, Colchester, UK
[8]University of Applied Sciences Western Switzerland (HES-SO), Switzerland
[*]corresponding author(s): Christoph M. Friedrich (christoph.friedrich@fh-dortmund.de), technical inquiries: Johannes Rückert (johannes.rueckert@fh-dortmund.de)
[†]contributed equally

Supplementary Information

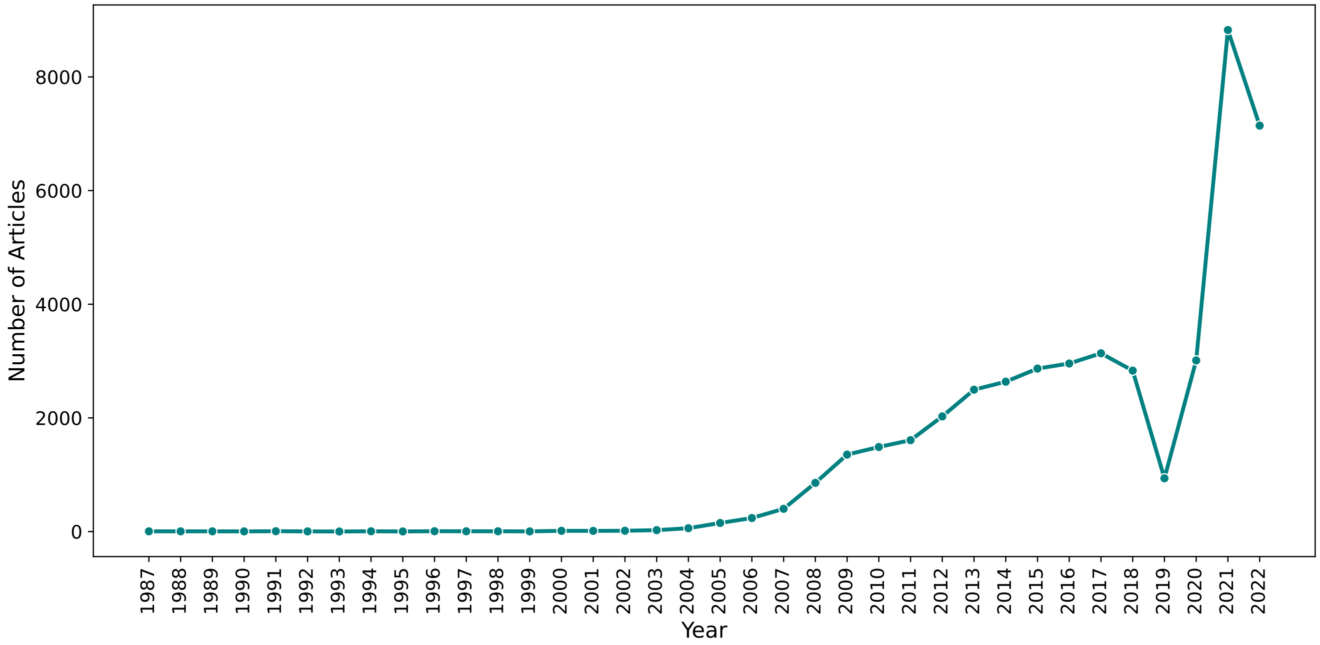

Figure S1. The distribution of article publications from 1987 to 2022, which were utilized for our data extraction. From 1987 to 2002, publications remained below 15 annually. A remarkable increase commenced in 2003, peaking in 2021 with 9341 articles. A dip in 2019 can be explained by regular updates to the dataset, which was originally released in 2018, beginning in 2020.

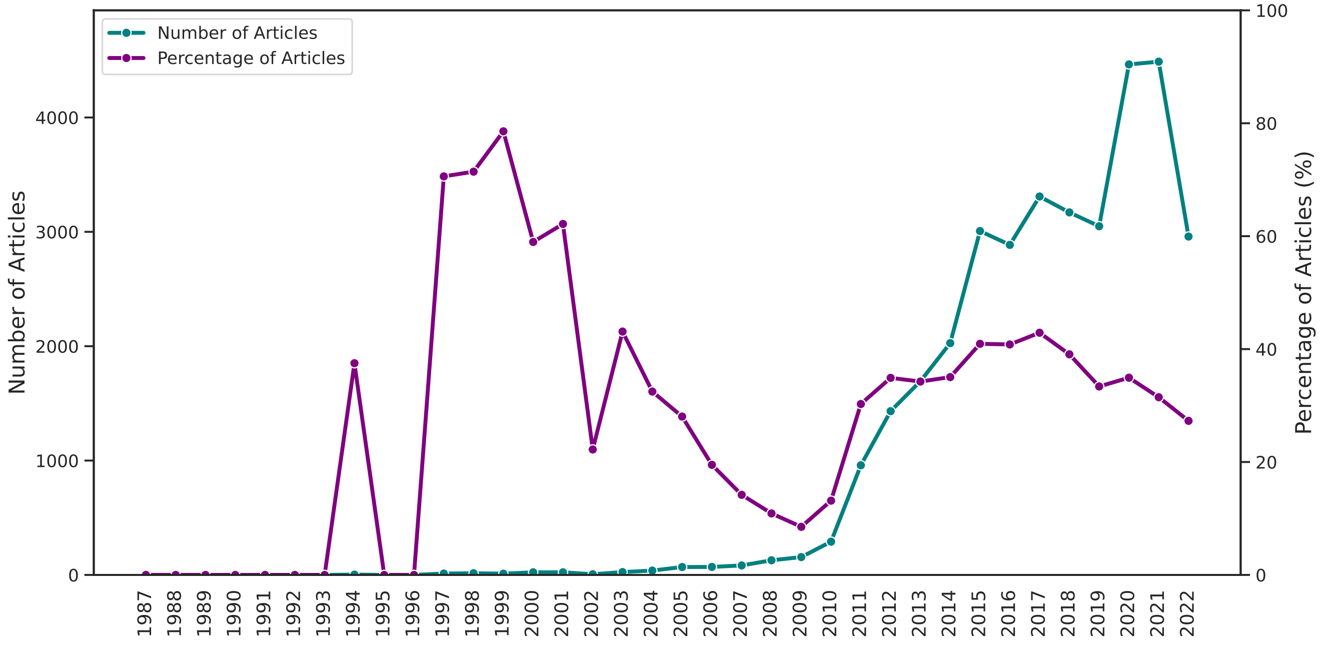

Figure S2. The relative and absolute distribution of CC BY-ND or CC BY-SA licensed PubMed articles from the years 1987 to 2022. Images from these articles were not considered for the dataset based on their license. When comparing the numbers with Figure S1, it can be seen that these licenses have become increasingly popular from 2011 with a peak in 2017.

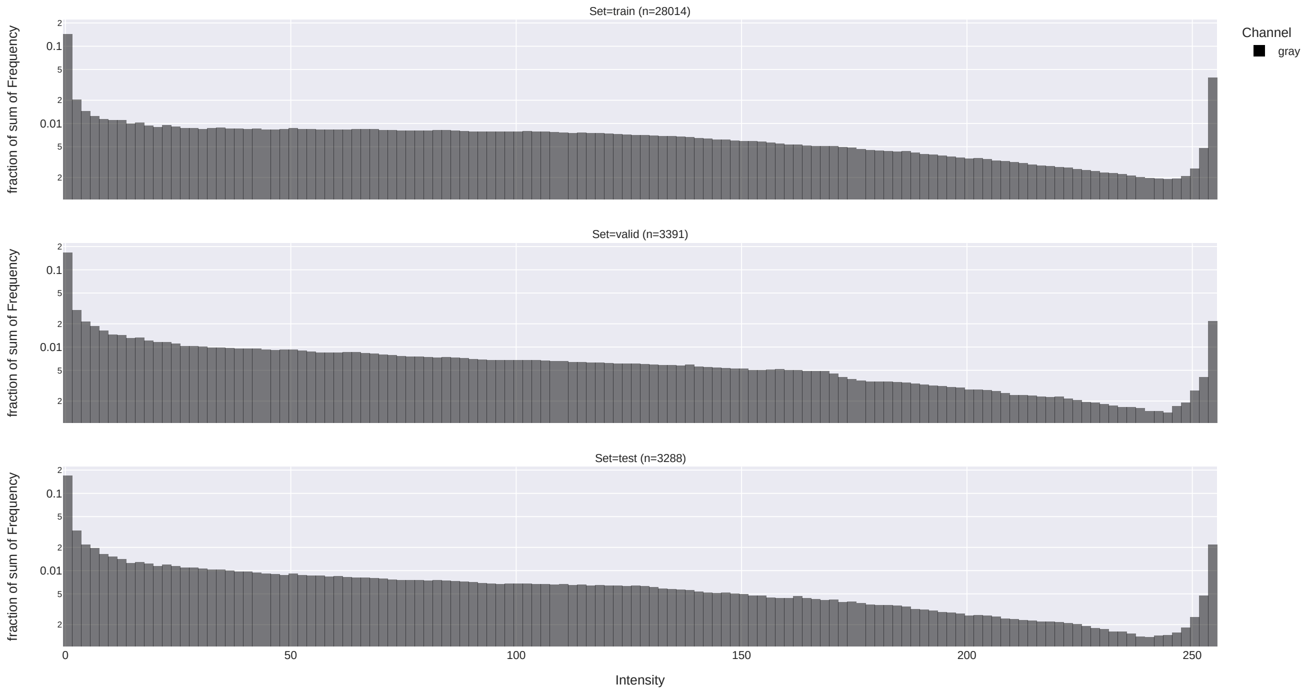

(a) Grayscale images ($n = 34,693$) intensity histograms (128 bins) of train, valid, and test sets.

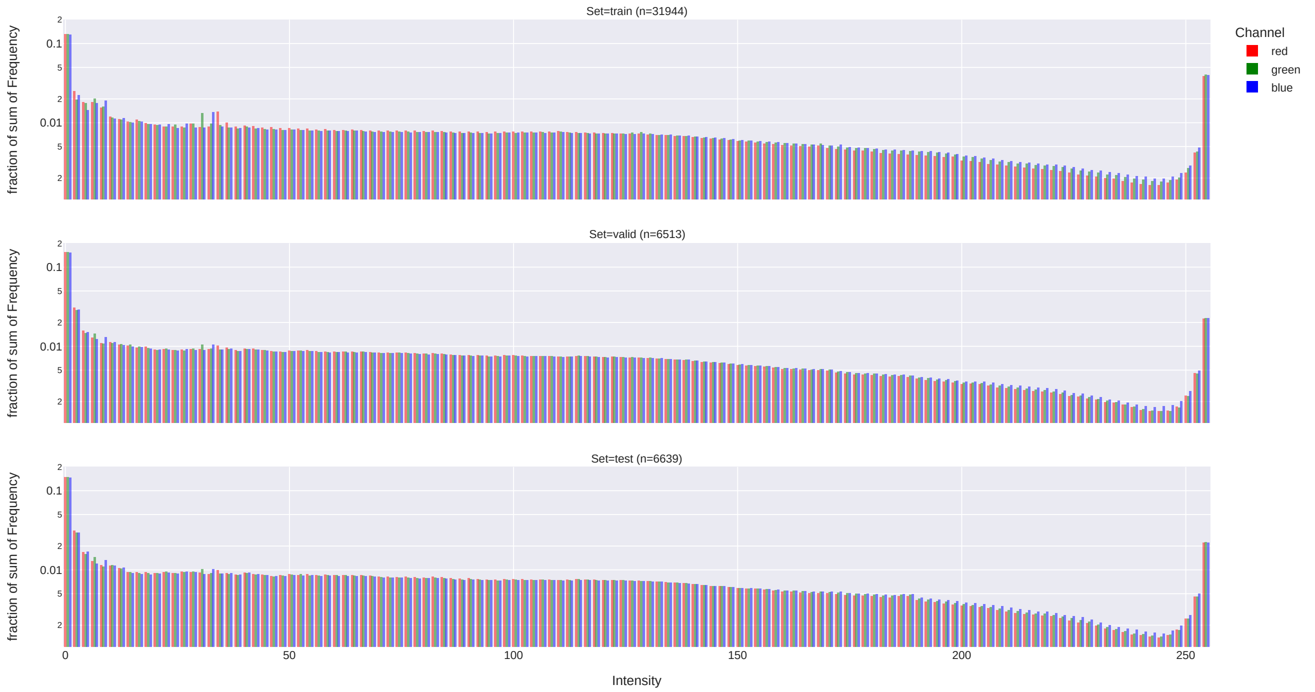

(b) RGB images ($n = 45,096$) intensity histograms (128 bins) of train, valid, and test sets.

Figure S3. Grayscale and RGB images ($n = 79,789$) intensity histograms of train, valid, and test sets.

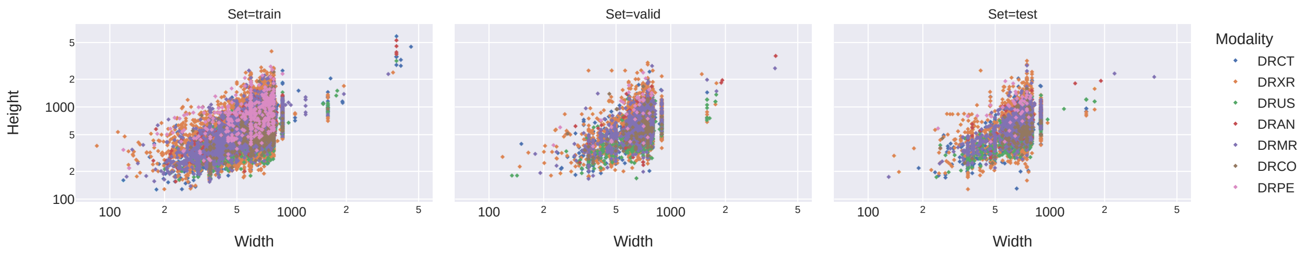

(a) Image width, height distributions of train, valid, test sets.

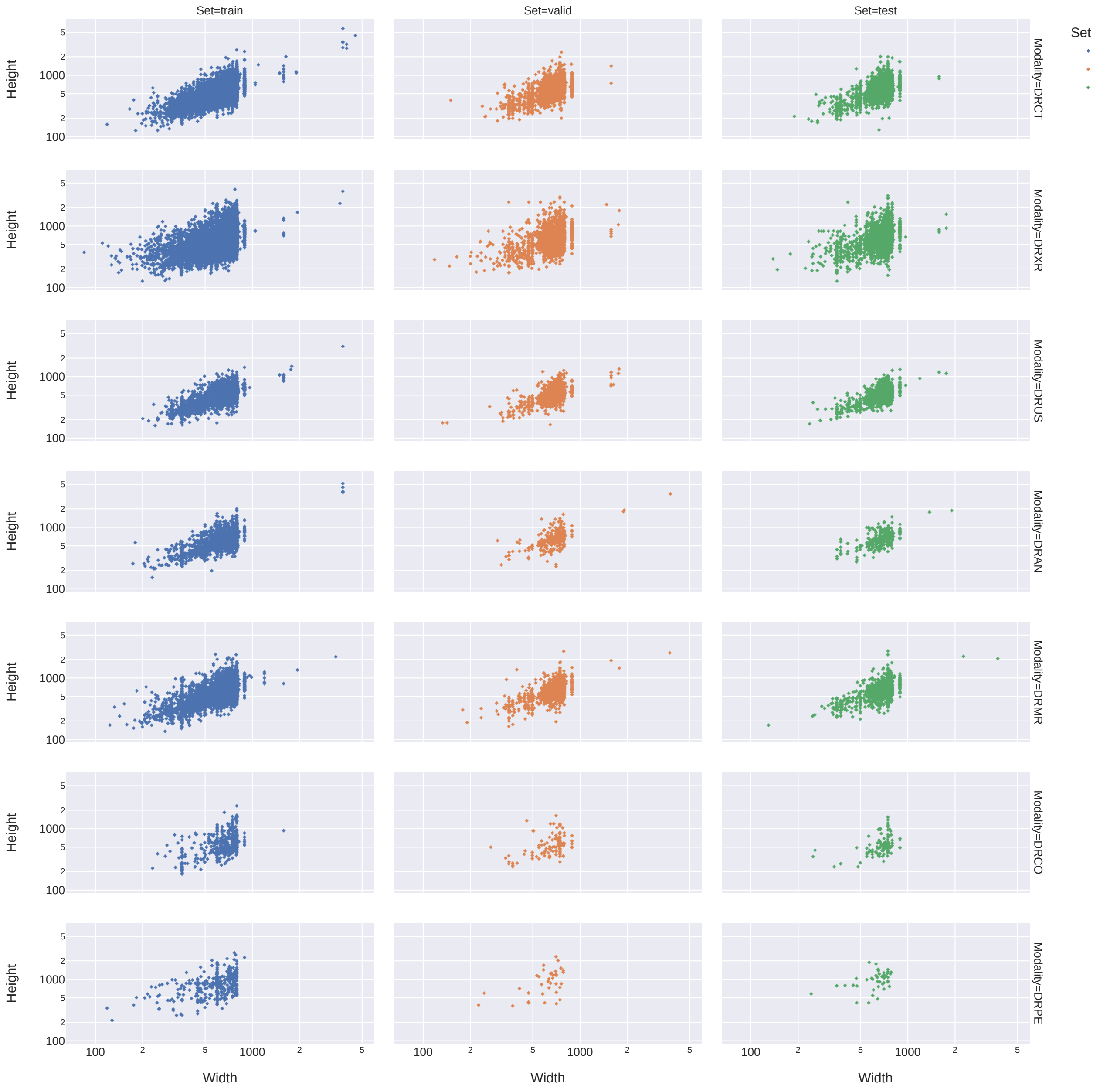

(b) Image width, height distributions of train, valid, test sets separated by modality.

Figure S4. Image width, height distributions.

Table S1. Frequency Analysis of Publication Types provided by PubMed. The table presents the publication type distribution of distinct PMC articles from which image/caption pairs have been extracted. Each article may be associated with multiple publication types. Note that the analysis is limited to those articles where publication types were explicitly available within the MEDLINE PubMed Citation database. 187 PMC articles were excluded due to not having standardized publication types available. Single publication type occurrences are omitted.

Publication Type	Frequency
Journal Article	26,757
Case Reports	26,474
Review	2991
Research Support, Non-U.S. Gov't	1546
Comparative Study	293
Observational Study	193
Letter	178
Evaluation Study	152
Randomized Controlled Trial	128
Research Support, N.I.H., Extramural	122
Clinical Trial	116
Multicenter Study	98
Systematic Review	65
Video-Audio Media	39
Retracted Publication	38
Validation Study	32
Editorial	30
Comment	26
Research Support, U.S. Gov't, Non-P.H.S.	23
Historical Article	17
Clinical Study	14
Meta-Analysis	12
Clinical Trial, Phase II	11
Clinical Trial Protocol	10
Practice Guideline	9
Controlled Clinical Trial	9
Research Support, N.I.H., Intramural	8
Research Support, U.S. Gov't, P.H.S.	6
Randomized Controlled Trial, Veterinary	5
English Abstract	5
Clinical Trial, Phase I	4
Published Erratum	4
Observational Study, Veterinary	4
Dataset	4
Clinical Conference	3
Congress	2
Technical Report	2
Clinical Trial, Phase III	2
Guideline	2
Equivalence Trial	2
**Total**	**59,446**

Table S2. Modality distribution of image and caption pairs within ROCOv2: Angiography modality (DRAN), combined modality (DRCO), CT modality (DRCT), MRI modality (DRMR), PET modality (DRPE), ultrasound modality (DRUS), and X-ray modality (DRXR).

Modality	Count
DRCT	27,747
DRXR	21,997
DRMR	12,657
DRUS	11,429
DRAN	4799
DRCO	728
DRPE	432
**Total**	**79,789**

Table S3. Distribution of manually created body part labels for the X-ray modality (DRXR).

IRMA body part	Count
chest	7388
cranium	3973
lower_extremity	3167
abdomen	2301
pelvis	1573
upper_extremity	1478
spine	1363
breast	102
**Total**	**21,345**

Table S4. Distribution of manually created directionality labels for the X-ray modality (DRXR).

Category	Count
coronal_ap	9605
coronal_pa	4303
sagittal	2425
transversal	51
**Total**	**16,384**

Table S5. Top 100 journal distribution of PMC articles used in ROCOv2.

Journal	PMC Articles	Journal	PMC Articles
Cureus	6249	Frontiers in Pediatrics	164
Journal of Medical Case Reports	1514	International Journal of Clinical Pediatric Dentistry	163
The Pan African Medical Journal	1396	Sage Open Medical Case Reports	153
Journal of Surgical Case Reports	1128	Yonsei Medical Journal	151
Medicine	782	Case Reports in Oncological Medicine	149
Clinical Case Reports	773	Journal of Korean Neurosurgical Society	146
Cases Journal	643	Case Reports in Pulmonology	145
Case Reports in Medicine	597	The Journal of International Medical Research	144
Case Reports in Dentistry	478	Imaging Science in Dentistry	141
World Journal of Surgical Oncology	473	BMC Research Notes	138
PLoS One	462	BMC Infectious Diseases	137
Case Reports in Surgery	451	Case Reports in Emergency Medicine	137
Case Reports in Orthopedics	426	Frontiers in Oncology	137
International Journal of Surgery Case Reports	383	Journal of Medicine and Life	134
Journal of Cardiothoracic Surgery	371	Frontiers in Surgery	134
World Journal of Clinical Cases	349	Journal of Orthopaedic Case Reports	130
Journal of Clinical Medicine	331	Journal of Community Hospital Internal Medicine Perspectives	129
Case Reports in Cardiology	317	The Korean Journal of Thoracic and Cardiovascular Surgery	127
Clinical Practice and Cases in Emergency Medicine	306	The Indian Journal of Radiology & Imaging	126
Case Reports in Obstetrics and Gynecology	301	Journal of the Belgian Society of Radiology	126
BMC Musculoskeletal Disorders	299	Korean Circulation Journal	125
European Heart Journal. Case Reports	295	Archives of Plastic Surgery	124
Diagnostics	294	World Journal of Emergency Surgery	121
BJR Case Reports	289	Iranian Journal of Radiology	121
Annals of Medicine and Surgery	257	International Journal of Environmental Research and Public Health	117
Surgical Case Reports	249	Clinics in Orthopedic Surgery	116
Case Reports in Urology	242	BMC Gastroenterology	115
Korean Journal of Radiology	230	Insights into Imaging	113
Scientific Reports	230	Cardiovascular Ultrasound	110
Case Reports in Otolaryngology	221	Case Reports in Critical Care	110
The Western Journal of Emergency Medicine	220	Case Reports in Endocrinology	107
Case Reports in Gastroenterology	219	Cardiology Research	107
Case Reports in Pediatrics	212	Radiology Case Reports	106
Oxford Medical Case Reports	211	Case Reports in Vascular Medicine	105
Korean Journal of Anesthesiology	210	Clinical Endoscopy	103
Journal of Korean Medical Science	206	Children	101
BMJ Case Reports	205	F1000Research	100
Case Reports in Infectious Diseases	202	Case Reports in Rheumatology	98
The Korean Journal of Internal Medicine	196	Frontiers in Medicine	97
Case Reports in Oncology	196	Journal of the Korean Association of Oral and Maxillofacial Surgeons	95
Journal of Orthopaedic Surgery and Research	190	Journal of Clinical Medicine Research	94
Case Reports in Gastrointestinal Medicine	188	Frontiers in Neurology	94
Medicina	185	Gastroenterology Research	91
BMC Surgery	183	BMC Cardiovascular Disorders	90
Radiologia Brasileira	183	Case Reports in Neurological Medicine	90
Case Reports in Radiology	179	Cancers	90
Journal of Investigative Medicine High Impact Case Reports	176	Journal of Clinical and Experimental Dentistry	90
BioMed Research International	170	Dental Press Journal of Orthodontics	90
Asian Spine Journal	166	Diagnostic Pathology	88
Oncology Letters	165	Case Reports in Pathology	88

Table S6. Top 10 frequent CUIs for the angiography modality (`DRAN`).

CUI	UMLS Term	Images
C0002978	Angiogram	4799
C0226032	Anterior Descending Branch Of Left Coronary Artery	516
C0038257	Stent, Device	399
C1261287	Stenosis	345
C1261316	Right Coronary Artery Structure	338
C0034052	Pulmonary Artery Structure	296
C1947917	Occluded	257
C0085590	Catheter Device	245
C0001168	Complete Obstruction	220
C0002940	Aneurysm	212

Table S7. Top 10 frequent CUIs for the combined modality (`DRCO`).

CUI	UMLS Term	Images
C0032743	Positron-Emission Tomography	232
C1699633	Pet/Ct Scan	208
C0034606	Radionuclide Imaging	74
C0027651	Neoplasms	56
C0040405	X-Ray Computed Tomography	51
C0024204	Lymph Nodes	50
C0028259	Nodule	36
C0036525	Metastatic To	30
C0023884	Liver	30
C2939419	Secondary Neoplasm	27

Table S8. Top 10 frequent CUIs for the CT modality (`DRCT`).

CUI	UMLS Term	Images
C0040405	X-Ray Computed Tomography	27,747
C0817096	Chest	2431
C0000726	Abdomen	1651
C0030797	Pelvis	1327
C0444611	Fluid Behavior	838
C0027651	Neoplasms	832
C0023884	Liver	715
C0205207	Cystic	661
C0028259	Nodule	595
C0225317	Soft Tissue	552

Table S9. Top 10 frequent CUIs for the MRI modality (`DRMR`).

CUI	UMLS Term	Images
C0024485	Magnetic Resonance Imaging	12,657
C0006104	Brain	724
C0027651	Neoplasms	486
C0013604	Edema	466
C0444611	Fluid Behavior	440
C0037925	Spinal Cord	357
C0205207	Cystic	332
C0030797	Pelvis	304
C0152295	Cerebral White Matter Structure	213
C0018787	Heart	211

Table S10. Top 10 frequent CUIs for the PET modality (`DRPE`).

CUI	UMLS Term	Images
C0032743	Positron-Emission Tomography	432
C0024204	Lymph Nodes	44
C0025066	Mediastinum	30
C0023884	Liver	27
C0027651	Neoplasms	25
C1266909	Entire Bony Skeleton	24
C0037993	Spleen	20
C0006826	Malignant Neoplasms	18
C0036525	Metastatic To	18
C0030797	Pelvis	18

Table S11. Top 10 frequent CUIs for the ultrasound modality modality (`DRUS`).

CUI	UMLS Term	Images
C0041618	Ultrasonography	11,429
C0225897	Left Ventricular Structure	764
C0225883	Right Ventricular Structure	611
C0225860	Left Atrial Structure	429
C0018827	Heart Ventricle	385
C0205207	Cystic	384
C0225844	Right Atrial Structure	361
C0003483	Aorta	331
C0018792	Heart Atrium	310
C0031039	Pericardial Effusion	301

Table S12. Top 10 frequent CUIs for the X-ray modality (`DRXR`).

CUI	UMLS Term	Images
C1306645	Plain X-Ray	21,997
C1999039	Anterior-Posterior	9605
C0817096	Chest	7535
C1996865	Postero-Anterior	4303
C0037303	Bone Structure Of Cranium	3987
C0023216	Lower Extremity	3183
C0205129	Sagittal	2430
C0000726	Abdomen	2375
C0030797	Pelvis	1845
C1140618	Upper Extremity	1478

Table S13. Distribution of non-English captions.

**Language**	**Number of Captions**	**Proportion (%)**
French	1413	92.47
Portuguese	55	3.60
Spanish	48	3.14
Dutch	4	0.26
German	4	0.26
Italian	2	0.13
Turkish	1	0.07
Russian	1	0.07
**Total**	**1528**	**100.00**

Table S14. Distilled annotation guideline for manual labeling of angiography (`DRAN`), combined (`DRCO`), CT (`DRCT`), MRI (`DRMR`), PET (`DRPE`), ultrasound (`DRUS`), and X-ray (`DRXR`) modalities.

**Label**	**Matching criteria**
General information:	For each image the given caption is to be checked when its modality can not be determined with very high certainty by its visual features. In case its caption does not provide sufficient information or is ambiguous, checking its context within its original publication is demanded. Images may comprise annotations, e.g., arrows, markers, or sketches, up to a limit where these are dominating the visual. Images with a veterinary focus are to be included.
`DRAN`	2D X-ray projections that show application of contrast media for the specific purpose of highlighting blood vessels, e.g., ventriculography, aortography, or arterio/-venography. Angiographic methods performed using CT/MRI are not to be considered as part of this class, but as CT modality (`DRCT`) respectively MRI modality (`DRMR`).
`DRCO`	Combination of supported modalities, e.g., PET-CT/MRI, or SPECT-CT/MRI. However, CT/MRI scans with angiographic components are not considered.
`DRCT`	Classic CT scan images, e.g., single slices, or accumulations of few slices. Images involving contrast media are considered regular CT scans, also those with angiographic components. Vast 3D representations rendered from CT scan volume information are not considered. Combined with other imaging techniques, e.g. PET, SPECT, or likewise, images need to be considered as combined modality (`DRCO`).
`DRMR`	Classic MRI scan images, e.g., single slices, or accumulations of few slices. Images involving contrast media are considered regular MRI scans, also those with angiographic components. Vast 3D representations rendered from MRI scan volume information are not considered. Combined with other imaging techniques, e.g. PET, SPECT, or likewise, images need to be considered as combined modality (`DRCO`).
`DRPE`	Solely classic PET scans. Other similar but not positron emission-based methods, e.g., SPECT, or scintigraphy, are not considered. PET scans combined with CT/MRI are have to be considered as combined modality (`DRCO`).
`DRUS`	Classic 2D/3D ultrasound images. These may also represent user interface screenshots or comprise additional data on, e.g., flow series, Doppler visualization, or location.
`DRXR`	Classic 2D X-ray projections, but also X-ray panorama shots of the jaw region. The latter need to be differentiated from Cone Beam CT (CBCT) shots that share a certain resemblance. Methods using contrast media, e.g., in Barium swallow, urethrography, arthrogram, or cholangiography, are considered regular X-ray projections. However, methods using contrast media to specifically highlight blood vessels have to be considered as angiography modality (`DRAN`).
`OTHER`	Label that pools diverse non-radiological and out-of-class images, later to be excluded from the raw dataset. This comprises compound images that incorrectly passed the compound filtering mechanism, vast 3D rendered images, synthetically generated images (e.g., by generative adversarial networks, or diffusion networks), photographs/sketches/collages (e.g., scenes, schematics, workflows, or multiple images of different contexts), non-medical modalities (e.g., transmission electron microscopy, or satellite images), non-supported modalities (e.g., optical coherence tomography (OCT), endoscopy, or histological slices), images representing a supported modality but not showing a specific medical context (e.g., assessment of materials, phantoms, or artifacts), images that contained captions as image parts and thus could leak information when using optical character recognition (OCR) techniques, and several others.
`UNKNOWN`	Label that pools images which can not be labeled with certainty due to insufficient information from visual features, captions, and publication context, later to be excluded from the raw dataset.

Table S15. Distilled annotation guideline for manual labeling of body regions for the X-ray modality (DRXR).

Label	Matching criteria
General information:	For each image the given caption is to be checked when its body region can not be determined with very high certainty by its visual features. In case its caption does not provide sufficient information or is ambiguous, checking its context within its original publication is demanded. Images may comprise annotations, e.g., arrows, markers, or sketches, up to a limit where these are dominating the visual. The given context described by the caption is relevant, images showing multiple regions may not be excluded if the given context demands an expanded projection (e.g., a pronounced scoliosis displayed over chest and abdomen regions actually focuses the spine). The given projection directionality is not relevant. Images with a veterinary focus are to be excluded, as the underlying IRMA classification solely addresses human anatomy.
abdomen	X-ray projections that cover a region ranging from the upper abdominal structures (e.g., diaphragma border, lower ribs, liver, stomach) down to the lower abdominal structures (e.g., bladder with urinary tract, gynecological organs, anus). A mild overlap with the chest region is acceptable, a moderate overlap may be acceptable given that the caption context addresses the abdomen region. A total overlap with the pelvis region is acceptable given the caption context clearly addresses the abdomen region.
breast	X-ray projections solely acquired from mammography.
chest	X-ray projections that cover a region from the upper thoracic structures (e.g., upper ribs, claviculae, upper pars thoracica of esophagus) down to the lower thoracic structures (e.g., thoracic diaphragma, lower ribs, lower lobes of lungs). A mild overlap with the cranium and abdomen regions is acceptable, a moderate overlap may be acceptable given that the caption context clearly addresses the chest region. Projections acquired via mammography are to be labeled as breast.
cranium	X-ray projections that mainly cover osseous cranial structures (e.g., cranium, mandibula, teeth). A mild overlap with the chest region is acceptable, a moderate overlap may be acceptable given that the caption context clearly addresses the cranium region.
lower_extremity	X-ray projections that cover a region from hip joints (excluding the acetabulum as part of the pelvic bone) down to the toes. A mild overlap with the lower abdomen respectively pelvis regions is acceptable, a moderate overlap may be acceptable given that the caption context clearly addresses the lower extremity region.
pelvis	X-ray projections that mainly cover the osseous pelvic structures (e.g., pelvic bone, sacrum, acetabulum). A mild overlap with the lower extremity region is acceptable, a moderate overlap may be acceptable given that the caption context addresses the pelvic region. A total overlap with the lower abdominal region is unavoidable, yet the given caption context must clearly address the pelvic structures that are not covered by abdominal structures.
spine	X-ray projections that cover a region from C1 of the cervical spine down to L5 of the lumbar spine. A total overlap with the cranium, chest, abdomen, and pelvis region is unavoidable, yet the given caption context must clearly address the spinal structures. Contrary to other classes, full-body or full-torso projections that would be considered OTHER due to excessive region overlaps may be acceptable to be labeled as spine for cases that clearly address the whole spine (e.g., angle measurements, pronounced scoliosis, osteoporosis diagnostics).
upper_extremity	X-ray projections that cover a region from glenohumeral joints down to the fingers. A mild overlap with the lower chest and abdomen regions is acceptable, a moderate overlap may be acceptable given that the caption context clearly addresses the upper extremity region.
OTHER	Label that pools mixed-class and out-of-class images, that stay within the dataset but do not receive a manual annotations for body regions. This comprises images that either display a moderate to heavy overlap between regions without a clear focus on a specific region (e.g., too wide projection angles, cases multimorbidity presented in a single projection, pediatric full-body checks), veterinary cases, or very rare cases that can not be classified with certainty into the IRMA system (e.g., lateral projections of outer genitalia, lateral Barium swallow for upper esophagus cavity diagnostics).
UNKNOWN	Label that pools images which can not be labeled with certainty due to insufficient information from visual features, captions, and publication context. These images stay within the dataset but do not receive a manual annotation.

Table S16. Distilled annotation guideline for manual labeling of directionality for the X-ray modality (DRXR).

Label	Matching criteria
General information:	For each image of the X-ray modality (DRXR), the directionality should be determined based on the standard exposures and projections. If the directionality is not clear from the image itself, additional context from the original publication or caption should be consulted. Images with a veterinary focus are to be excluded.
coronal_ap	Images that are taken in the coronal plane and are anteroposterior (AP) projections. This includes coronal chest X-rays taken with a portable device and in a supine or semi-erect position (e.g. emergency or bedside imaging) as well as standard coronal projections of the abdomen, spine, bones and joints of the pelvis, lower extremity, upper extremity (excluding hand and wrist) and, in some instances, the skull.
coronal_pa	Images that are taken in the coronal plane and are posteroanterior (PA) projections. This includes coronal chest X-rays taken in a standing position, as well as standard coronal projections of the hand and wrist, skull, and, in some instances, the lumbar spine.
sagittal	Images that are taken in the sagittal plane, showing a lateral view of the body part. This includes lateral views of the chest, spine, bones and joints of the upper and lower extremities and, in some instances, abdomen.
transversal	Images that are taken in the transversal (axial) plane. This includes axial projections of the shoulder and the knee.
OTHER	Label that pools mixed-class and out-of-class images, that stay within the dataset but do not receive a manual annotations for directionalities. This includes non-standard projections or views, such as dental X-rays (e.g., panoramic, bitewing, or periapical X-rays), special views of the skull (e.g., occipitofrontal, occipitomental, bregmaticooccipital, submentobregmatical, submentobregmaticofrontal, bregmaticooral, bregmaticosubmental), oblique angles (e.g., right anterior oblique (RAO), left anterior oblique (LAO)), or a combination of two projections within the same image.
UNKNOWN	Label that pools images which can not be labeled with certainty due to insufficient information from visual features, captions, and publication context. These images stay within the dataset but do not receive a manual annotation.
